# Reliability and sensitivity to change of post-match physical performance measures in elite youth soccer players

**DOI:** 10.3389/fspor.2023.1173621

**Published:** 2023-07-13

**Authors:** Alberto Franceschi, Mark A. Robinson, Daniel Owens, Thomas Brownlee, Duccio Ferrari Bravo, Kevin Enright

**Affiliations:** ^1^ Sport Science and R&D Department, Juventus Football Club, Torino, Italy; ^2^ School of Sport and Exercise Sciences, Liverpool John Moores University, Liverpool, United Kingdom; ^3^ School of Sport, Exercise and Rehabilitation Sciences, University of Birmingham, Birmingham, United Kingdom

**Keywords:** monitoring, neuromuscular fatigue, recovery, reliability, responsiveness, football

## Abstract

**Introduction:**

To effectively monitor post-match changes in physical performance, valid, reliable and practical measures which are sensitive to change are required. This study aimed to quantify test-retest reliability and sensitivity to change of a range of physical performance measures recorded during an isometric posterior chain (IPC) lower-limb muscle test and a countermovement jump (CMJ) test.

**Methods:**

Eighteen Italian Serie A academy soccer players performed three IPC repetitions per limb and five CMJ trials in 4 testing sessions. Test-retest reliability was evaluated between two testing sessions seven days apart using typical error of measurement, coefficient of variation and intraclass correlation coefficient. Sensitivity to change was assessed on two additional testing sessions performed before and immediately after a soccer match through Hedges' g effect size (g) and comparisons to typical error.

**Results:**

Absolute reliability (coefficient of variations) ranged from 1.5 to 8.8%. IPC and CMJ measures demonstrated *moderate* to *excellent* relative reliability (intraclass correlation coefficients ranged from 0.70 to 0.98). A wide range of physical performance measures showed significant alterations post-match (*p* < 0.05; *g*: *small* to *moderate*). IPC peak force and torque, CMJ reactive strength index modified, CMJ eccentric forces (mean breaking force, mean deceleration force, peak force, force at zero velocity) and CMJ mean power measures had post-match changes greater than their typical variation, demonstrating acceptable sensitivity in detecting performance changes at post-match.

**Discussion:**

IPC peak force and torque, CMJ reactive strength index modified, CMJ eccentric phase forces and CMJ mean power were found to be both reliable and sensitive to change, and thus may be appropriate for monitoring post-match neuromuscular performance in youth soccer population.

## 1. Introduction

Soccer match-play represents the most demanding stimulus of the competitive microcycle (1, 2) and has been shown to induce metabolic and mechanical fatigue acutely and in the days following the match in elite senior and youth soccer players (3–6). Nowadays, elite youth soccer players can be exposed to demanding periods of training and competition with limited between-match recovery time (7). The collective stresses accumulated during the competitive season may lead players to a fatigued status, which may impact their preparedness for subsequent competition and increase the risk of non-functional overreaching, injury, and illness (8). This scenario has prompted a greater interest in monitoring players' post-match fatigue to inform training loads and recovery strategies within the weekly microcycle (9). Fatigue can be defined as a decline in an objective measure of performance over a discrete period (e.g., a decrease in force and power output), and as an increased perception of effort that regulates the integrity of an individual (10). A myriad of field-based physical performance tests is used to assess individual responses following training or competition (11), including isometric tests (e.g., squat, mid-thigh pull, posterior chain) and dynamic measures such as vertical jumps (12). Despite the widespread use of these player monitoring tools, evidence regarding the suitability of a comprehensive range of physical performance measures for monitoring post-match responses in a youth soccer population is limited.

A suitable measure used for effectively monitoring post-match response should possess high reproducibility between repeated tests performed under similar conditions (i.e., reliability) (13, 14) and should be capable of detecting changes induced by training or a match (i.e., sensitivity to change or responsiveness) (9). The reliability of a test refers to the degree of consistency between repeated tests within a practically relevant timeframe (13, 14), while sensitivity to change refers to the ability of a measure to change over a particular timeframe (15). Quantifying both test-retest reliability and sensitivity to change of post-match neuromuscular fatigue measures has been recommended to better interpret individual responses in high-performance sports (16, 17). Additionally, considering the challenges of team-sport settings, tests used for post-match response monitoring should be selected according to their relevance to performance and feasibility within the environment (18). Although numerous studies have reported the reliability of physical performance measures in youth soccer players including vertical jumps and isometric tests (19–21), less research has focused on examining their sensitivity to change following match-play (22).

The countermovement jump (CMJ) test is widely used in high-performance sports to indirectly monitor changes in neuromuscular fatigue and subsequent recovery in both team and individual sports (23, 24). Previous research in youth soccer has mainly investigated the reliability of outcome measures such as jump height (CVs: 4.3%–4.8%; ICCs: 0.83–0.88) (19, 20). Despite possessing acceptable levels of reliability, jump height has been demonstrated to have limited sensitivity to change in training loads in youth and senior soccer players (25, 26). Following a youth academy match play, decrements in CMJ height were observed immediately post and at +24 h, with a tendency towards recovery at +48 h post-match (6). Considering the metabolic, mechanical and neural factors associated with neuromuscular fatigue (10), measuring ground reaction forces during the CMJ may offer superior insights for indirectly detect neuromuscular fatigue (27). Previous research has examined the reliability and sensitivity to change of various CMJ measures in other sports, demonstrating their suitability for ongoing monitoring (16, 22, 28, 29). In the context of youth soccer, additional research is required to identify reliable CMJ measures capable of detecting neuromuscular fatigue changes.

In team sports, physical performance monitoring also includes isometric tests, used in combination with vertical jumps (12) or as an alternative evaluation (30). Predominantly due to the elevated high-speed running demands (31) and the role of fatigue on hamstring strain injuries in soccer (32), hamstring assessment protocols were introduced as a tool to quantify muscle-specific neuromuscular fatigue during the competitive season. Soccer match results in substantial muscle function impairments until 48–72 h post-match with alterations in muscle contractile properties and central motor output (3). Larger and longer magnitudes of alterations have been observed for knee flexors compared to knee extensors, suggesting the importance of monitoring hamstring function at post-match (33). An isometric posterior chain (IPC) test has been proposed to measure the peak forces generated by the posterior chain musculature at 30° and 90° knee flexion in professional soccer players (34). IPC peak forces have been demonstrated to be reliable (CVs: 4%–6%; ICCs: 0.82–0.95) (34, 35) and sensitive to change following a competitive match-play (relative post-match changes from −20% to −10%) (34, 36). Despite its applicability, peak force does not account for individual differences in limb length and research examining the peak torque generated from the maximal voluntary contraction during the IPC is lacking. Therefore, it is important to demonstrate the reliability and sensitivity of IPC peak force and torque in the specific population to inform practitioners of the potential applications of this testing protocol.

This study extends previous research by quantifying the measurement characteristics of a range of physical performance measures used for monitoring post-match responses in elite youth soccer. Monitoring post-match neuromuscular fatigue using reliable and sensitive measures could potentially inform training prescription and recovery strategies at the individual level within the current or upcoming microcycle to ultimately optimize players’ preparedness and development. Therefore, the aim of this study was to (1) quantify the reliability and (2) examine the sensitivity of a range of different physical performance measures from the IPC lower-limb muscle test and the CMJ test in a group of elite youth soccer players.

## 2. Materials and methods

### 2.1. Subjects

Eighteen elite youth soccer players (mean ± SD, age: 16.7 ± 0.3 years, height: 177.5 cm ± 5.9 cm, body mass: 70.4 ± 4.4 kg, percentage adult height: 99.5 ± 0.5%, maturity offset: 2.6 ± 0.5 years) from the under-17 squad of a professional soccer academy in the Italian Serie A participated in this study. All players were free from injury and illness and completed on average 10 h of training (i.e., 5 training sessions) per week, plus a competitive match. Data collection was part of the club's monitoring system and written informed consent was provided by parents or legal guardians. Ethics approval for the study was provided by the University Research Ethics Committee (22/SPS/006) and was conducted in accordance with the Declaration of Helsinki.

### 2.2. Design

**Figure 1:**
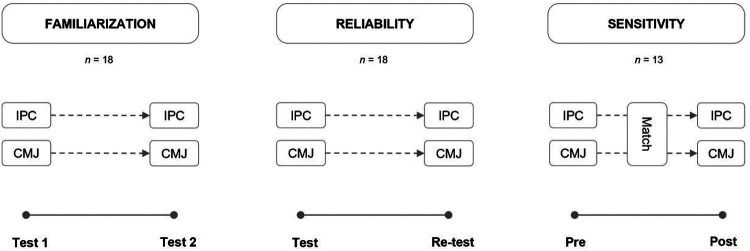
A schematic representation of the study design. Following the familiarization sessions, reliability was evaluated using a test-re-test design seven days apart. Sensitivity to change was evaluated following a match with testing sessions performed before and immediately after. IPC: isometric posterior-chain; CMJ: countermovement jump.

The study was completed during the competitive season and consisted of a test-retest reliability and sensitivity to change assessment of physical performance measures commonly used for post-match monitoring (Figure 1). The IPC and the CMJ test were used for all test sessions. All players received a minimum of two familiarization sessions in the weeks before the experimental trials. Reliability assessment consisted of two testing sessions spaced 7 days apart using a test-retest design. Test-retest reliability sessions were conducted following a 72 hour rest period during which players refrained from participating in training and vigorous activity Sensitivity to change was evaluated using an intra-club friendly match (2 × 40 min, 105 m × 68 m, artificial turf) as the intervention. Match demands were quantified using global navigation satellite systems (Apex Pro Series, 10 Hz GNSS, STATSports, Northern Ireland) and heart rate (HR) sensors (Polar H10, Polar, Finland). The average match demands were: total distance covered: 10,091 m ± 834 m; distance covered >20 km/h: 720 m ± 203 m; distance covered >25 km/h: 159 m ± 91 m; number of accelerations >3 m/s^2^: 60 ± 14; number of decelerations <−3 m/s^2^: 69 ± 15; time spent >85% HRmax: 29:48 ± 16:27 min; and time spent >90% HRmax: 9:13 ± 6:20 min. Each testing session was conducted at the same time of the day (i.e., early afternoon) to limit the influence of circadian variation and took place following a standardized 5-minute warm-up, except for the session performed immediately after the match. Testing sessions for sensitivity were performed pre- (i.e., 1 h and half before the commencement of the match) and immediately post-match (i.e., within 30 min of completing the match), including only the players who completed the full 80-minute duration. Players were instructed to maintain their normal dietary intake throughout the experimental period. Eighteen players completed the tests for reliability, while thirteen players completed the entire match and performed the tests for sensitivity.

### 2.3. Methodology

The IPC and the CMJ test were conducted using a force platform (ForceDecks Dual Force Plate System FD lite, VALD Performance, Australia). ForceDecks software was used to analyze and calculate the selected physical performance measures. The sampling rate of the force plate was 1,000 Hz.

#### 2.3.1. Isometric posterior chain (IPC) lower-limb muscle test

Players lay in a supine position with their knee at 90° (IPC-90°) or 30° (IPC-30°) of flexion, with their calcaneus on the center of the force platform and the non-test leg extended alongside a box at an appropriate height for each participant (i.e., lower shank to be parallel to the floor) (32). Players performed a 3s maximal voluntary contraction by driving their heel down as hard as possible into the platform. The tester ensured a correct position of both legs and pressure was applied to the contralateral hip to control participant posture (i.e., keeping the buttocks, hips and head on the floor). Players were required to repeat trials if their hips raised off the ground. Three trials on each limb were executed with 30s rest between trials for both IPC-90° and IPC-30° lower-limb muscle tests. Peak force was quantified for each trial. Moment arm length was measured from the joint axis of rotation to the point of application of the force and peak torque was calculated by multiplying the peak force by the length of the moment arm. For both the dominant and non-dominant leg, the three-trial average of peak force and peak torque were used for the subsequent statistical analysis.

#### 2.3.2. Countermovement jump (CMJ) test

Players performed five CMJ trials with 20s rest between trials. Before the commencement of each jump, players were advised to stand upright, with their arms akimbo and their feet hip-shoulder width apart. Once the starting position was adopted, players remained as still as possible for at least 3s before the start of the trial for the collection of the player's body weight. During the countermovement players were instructed to rapidly squat to their preferred depth and immediately jump as fast and as high as possible, with no knee or hip flexion during the flight phase, maintaining the hands on the hips. Finally, players were encouraged to “absorb” the landing by flexing at the hips, knees, and ankles after impacting the force platform (37). The average of the three best trials was used for the subsequent statistical analyses (38) for the following CMJ measures: jump height (JH), contraction time (CT), reactive strength index modified (RSImod), concentric duration (ConcDur), concentric peak force (ConcPF), concentric peak velocity (ConcPV), concentric mean power (ConcMP), peak power (PP), eccentric duration (EccDur), eccentric braking phase duration (EccBrakPhDur), eccentric deceleration phase duration (EccDecPhDur), eccentric mean braking force (EccMBrakF), eccentric mean deceleration force (EccMDecF), eccentric peak force (EccPF), force at zero velocity (F@0 V) and eccentric mean power (EccMP) (Supplementary Material). These measures were selected to describe both outcome and movement strategy measures (28) and agree with previous works completed in other high-performance environments (16, 17).

### 2.4. Statistical analysis

Descriptive data are reported as mean ± standard deviation (SD). The assumption of normality was assessed using a Shapiro-Wilk test. Differences between the two reliability test sessions and between pre- and post-match for sensitivity were tested for each measure using a paired *t*-test with alpha ≤0.05. For the reliability analysis, the typical error (TE) of measurement and the coefficient of variation (CV) were reported as measures of absolute reliability, while the intraclass correlation coefficient 3,1 (ICC; 2-way mixed-effects) with 95% confidence intervals was reported as a measure of relative reliability (13, 39). ICCs were interpreted in line with the lower 95% CI boundary based on previous recommendations: *excellent* (>0.90), *good* (0.75–0.90), *moderate* (0.50–0.75) and *poor* (<0.50) (40). For the sensitivity to change analysis, the magnitude of differences was assessed using Hedges' *g* effect size (*g*) with 95% CI. Criteria for effect size statistics were interpreted as follows: *trivial* (<0.2), *small* (0.2–0.6), *moderate* (0.6-1.2), *large* (1.2–2.0) and *very large* (>2.0) (41). To determine the ability of each measure in detecting relevant post-match variations, changes were reported and assessed against TE values. If post-match changes were greater than 1.5 times the TE in the test, changes were deemed as sensitive to change (i.e., responsive) (13). The statistical analysis was performed using SPSS statistical software (IBM SPSS version 26, Chicago, IL, USA).

## 3. Results

### 3.1. Reliability of physical performance measures

Reliability statistics are shown in Table 1. Absolute reliability (CVs) ranged from 1.5 to 8.8%. Relative reliability (ICCs) ranged from *good* to *excellent* for IPC measures (ICC ≥0.93; 95% CI: 0.79 to 0.99), whilst it ranged from *moderate* to *excellent* for the CMJ measures (ICC ≥0.85; 95% CI: 0.61 to 0.99), except for Eccentric Braking Phase Duration (EccBrakPhDur) and Eccentric Deceleration Phase Duration (EccDecPhDur) (ICC: 0.70 and 0.77; 95% CI: 0.37 to 0.88, and 0.51 to 0.91, respectively).

**Table 1:** Reliability statistics of physical performance measures from the isometric posterior chain (IPC) and the countermovement jump (CMJ) test in elite youth soccer players.

	Test 1 Mean (SD)	Test 2 Mean (SD)	*t* test (p)	Change in mean (95% CI)	TE	CV	ICC (95% CI)
Isometric posterior chain test							
Peak Force (N)—Dominant leg at 90°	317.2 (54.5)	319.9 (57.1)	0.406	−2.7 (−9.6 to 4.1)	9.7	3.0	0.97 (0.92 to 0.99)
Peak Force (N)—Non-dominant leg at 90°	289.9 (49.2)	296.5 (47.2)	0.123	−6.6 (−15.3 to 2.0)	12.3	4.2	0.93 (0.84 to 0.97)
Peak Torque (Nm)—Dominant leg at 90°	128.3 (23.3)	129.6 (24.1)	0.307	−1.3 (−3.9 to 1.3)	3.7	2.9	0.98 (0.93 to 0.99)
Peak Torque (Nm)—Non-dominant leg at 90°	117.4 (21.7)	120.4 (20.6)	0.081	−3.0 (−6.3 to 0.4)	4.8	4.0	0.95 (0.89 to 0.98)
Peak Force (N)—Dominant leg at 30°	299.2 (49.1)	299.4 (45.8)	0.966	−0.2 (−9.8 to 9.4)	13.6	4.6	0.92 (0.79 to 0.97)
Peak Force (N)—Non-dominant leg at 30°	268.9 (47.2)	275.1 (45.6)	0.174	−6.2 (−15.3 to 3.0)	13.0	4.8	0.92 (0.80 to 0.97)
Peak Torque (Nm)—Dominant leg at 30°	121 (21.1)	121.1 (19.6)	0.965	−0.1 (−4.0 to 3.8)	5.6	4.6	0.93 (0.81 to 0.97)
Peak Torque (Nm)—Non-dominant leg at 30°	108.8 (19.7)	111.2 (20.1)	0.155	−2.7 (−6.4 to 1.1)	5.4	4.9	0.93 (0.82 to 0.97)
Countermovement jump test							
Jump Height (cm)	37.0 (3.9)	36.8 (3.7)	0.437	0.2 (−0.4 to 0.9)	1.0	2.6	0.93 (0.83 to 0.97)
Contraction Time (ms)	713.6 (73.1)	719.6 (84.6)	0.570	−6.0 (−27.7 to 15.8)	30.9	4.3	0.85 (0.64 to 0.94)
RSImodified (m/s)	0.53 (0.08)	0.52 (0.09)	0.296	0.01 (−0.01 to 0.03)	0.02	4.0	0.93 (0.83 to 0.97)
Concentric Duration (ms)	260.9 (28.7)	262.4 (34.7)	0.702	−1.5 (−9.9 to 6.8)	11.9	4.5	0.86 (0.66 to 0.95)
Concentric Peak Force (N)	1,757.4 (204.1)	1,749.7 (238.5)	0.693	7.7 (−32.8 to 48.2)	57.6	3.3	0.93 (0.83 to 0.97)
Concentric Peak Velocity (m/s)	2.77 (0.13)	2.77 (0.14)	0.578	0.01 (−0.02 to 0.04)	0.04	1.5	0.91 (0.77 to 0.96)
Concentric Mean Power (W)	2,087.5 (350.0)	2,083.6 (398.0)	0.847	3.8 (−37.4 to 45.1)	58.6	2.8	0.97 (0.94 to 0.99)
Peak Power (W)	3,702.6 (651.1)	3,723.0 (682.8)	0.524	−20.4 (−86.5 to 45.7)	94.0	2.5	0.98 (0.95 to 0.99)
Eccentric Duration (ms)	455.2 (48.6)	457.1 (54.8)	0.764	−2.0 (−15.6 to 11.6)	19.3	4.2	0.86 (0.67 to 0.95)
Eccentric Braking Phase Duration (s)	0.28 (0.44)	0.28 (0.43)	0.875	0.00 (−0.01 to 0.02)	0.02	7.6	0.77 (0.51 to 0.91)
Eccentric Deceleration Phase Duration (s)	0.16 (0.27)	0.16 (0.28)	0.584	0.00 (−0.01 to 0.01)	0.01	8.8	0.70 (0.37 to 0.88)
Eccentric Mean Braking Force (N)	896.8 (91.5)	900.5 (95.2)	0.614	−3.7 (−19.0 to 11.6)	21.8	2.4	0.95 (0.86 to 0.98)
Eccentric Mean Deceleration Force (N)	1,348.5 (142.8)	1,335.5 (167.1)	0.440	13.0 (−21.7 to 47.7)	49.4	3.7	0.90 (0.75 to 0.96)
Eccentric Peak Force (N)	1,739.9 (192.6)	1,737.2 (230.5)	0.926	−2.7 (−42.9 to 48.2)	64.8	3.7	0.91 (0.77 to 0.96)
Force at Zero Velocity (N)	1,729.5 (194.6)	1,725.5 (236.4)	0.903	4.0 (−42.6 to 50.6)	66.3	3.8	0.91 (0.77 to 0.97)
Eccentric Mean Power (W)	498.1 (76.6)	498.8 (61.6)	0.860	−0.7 (−16.8 to 15.4)	22.8	4.6	0.89 (0.73 to 0.96)

CI, confidence intervals; TE, typical error of measurement; CV, coefficient of variation; ICC, intraclass correlation coefficient, RSI, reactive strength index.

### 3.2. Sensitivity of physical performance measures

**Figure 2:**
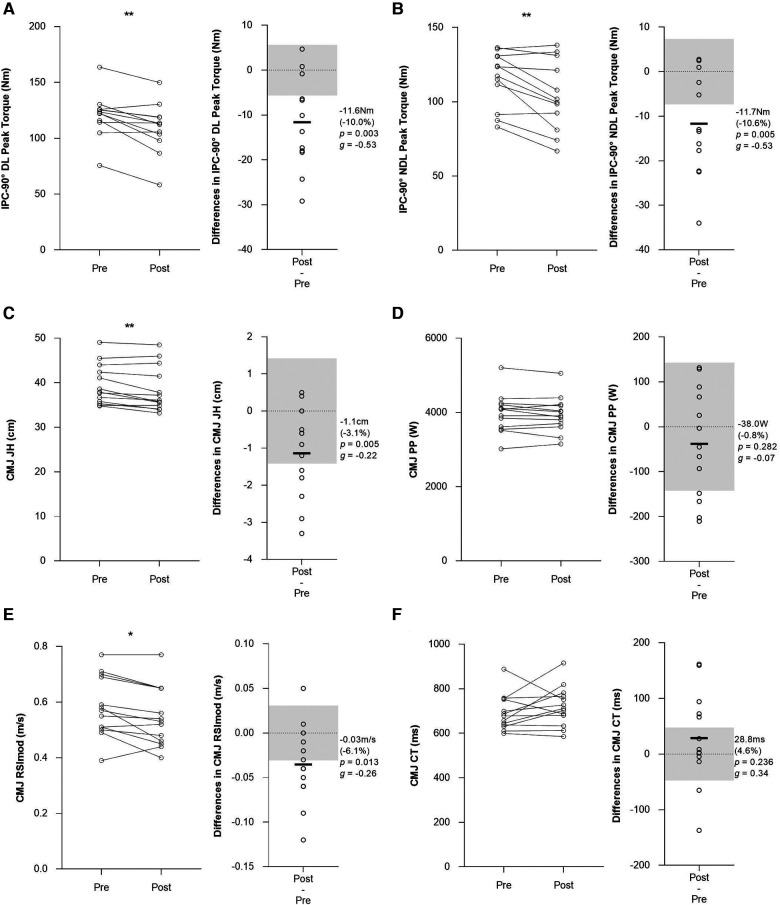
Post-match individual changes, mean difference (absolute and relative), *p*-value and Hedges’ *g* effect size of physical performance measures in elite youth soccer players. (**A**) IPC-90° peak torque of dominant leg, (**B**) IPC-90° peak torque of non-dominant leg, (**C**) CMJ jump height, (**D**) CMJ peak power, (**E**) CMJ RSImodified, (**F**) CMJ contraction time. Light grey boxes represent the 1.5 times the TE of each measure to identify the players who exhibited changes greater than the typical variance in the IPC and CMJ test. * indicates a significant mean difference at post-match (*p* ≤ 0.05), ** indicates a significant mean difference at post-match (*p* ≤ 0.01), ** indicates a significant mean difference at post-match (*p* ≤ 0.001). IPC-90° DL: isometric posterior chain 90° dominant leg; IPC-90° NDL: isometric posterior chain 90° non-dominant leg; JH: jump height; PP: peak power; RSImod: reactive strength index modified; CT: contraction time.

**Figure 3:**
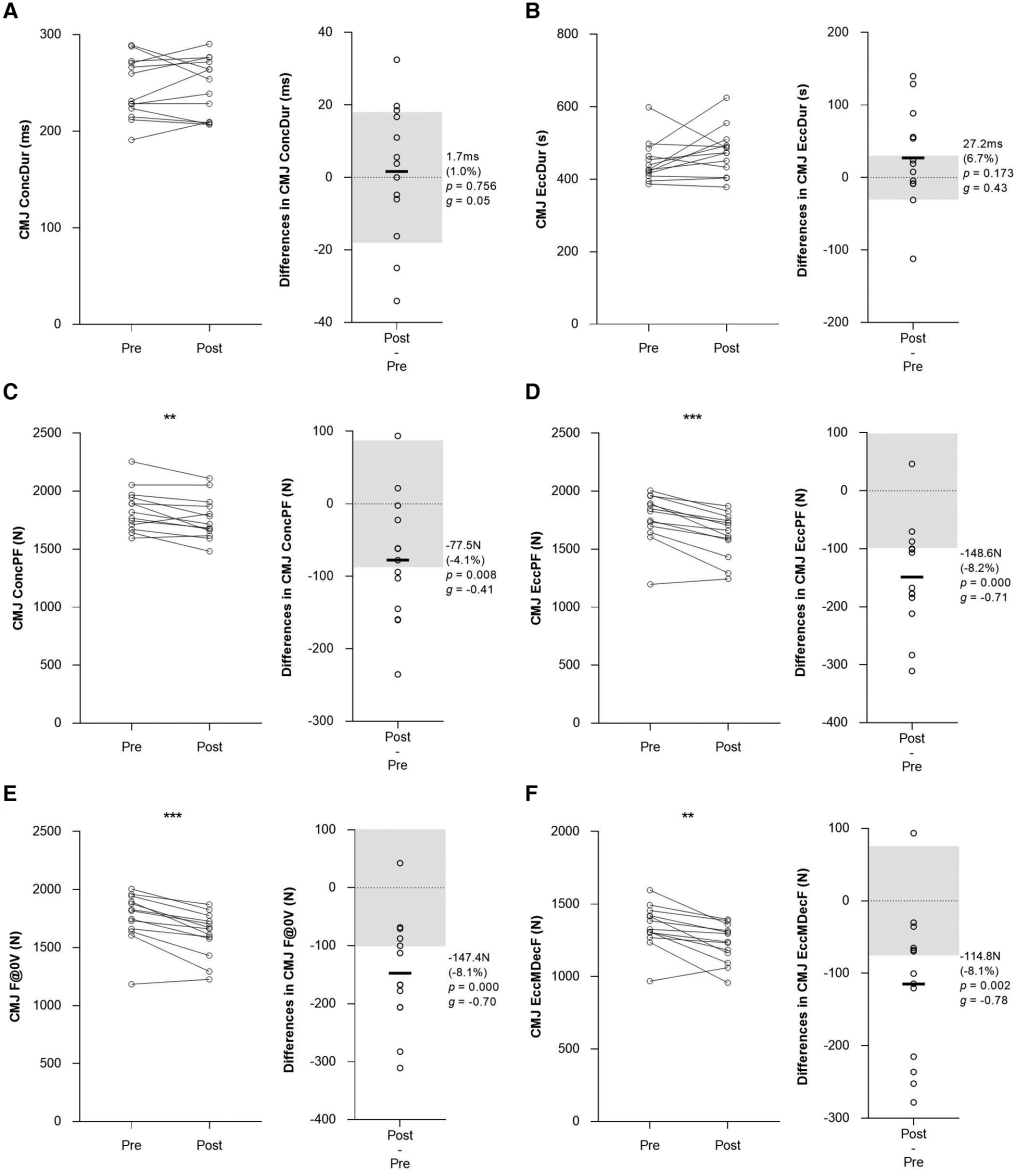
Post-match individual changes, mean difference (absolute and relative), *p*-value and Hedges’ *g* effect size of physical performance measures in elite youth soccer players. (**A**) CMJ concentric duration, (**B**) CMJ eccentric duration, (**C**) concentric peak force, (**D**) eccentric peak force, (**E**) force at zero velocity, (**F**) eccentric mean deceleration force. Light grey boxes represent the 1.5 times the TE of each measure to identify the players who exhibited changes greater than the typical variance in the IPC and CMJ test. * indicates a significant mean difference at post-match (*p* ≤ 0.05), ** indicates a significant mean difference at post-match (*p* ≤ 0.01), ** indicates a significant mean difference at post-match (*p* ≤ 0.001). ConcDur: concentric duration; EccDur: eccentric duration; ConcPF: concentric peak force; EccPF; eccentric peak force; F@0V: force at zero velocity; EccMDecF: eccentric mean deceleration force.

A wide range of IPC and CMJ measures showed significant alterations at post-match (*p* < 0.05) (Figures 2, 3, Table 2). The magnitude of change at post-match was *moderate* (from −10.0% to–10.6%; *g*: from −0.58 to −0.53) for IPC measures, while it spanned from *trivial* to *moderate* (from −0.8 to −10.7%; *g*: from −0.78 to 0.65) for CMJ measures (Figures 2, 3, Table 2). Eleven measures (IPC-90° PF DL, IPC-90° PF NDL, IPC-90° PT DL, IPC-90° PT NDL, RSImod, EccMBF, EccMDecF, EccPF, F@0 V, EccMP, ConcMP) displayed mean changes greater than typical variation (i.e., 1.5 times the TE) (Figures 2, 3, Table 2). Conversely, the other nine measures (JH, CT, ConcPF, EccDur, EccBrakPhDur, EccDecPhDur, ConcDur, ConcPV, PP) displayed mean changes lower than their corresponding typical variation (Figures 2, 3, Table 2).

**Table 2:** Sensitivity to change of physical performance measures from the isometric posterior chain (IPC) and the countermovement jump (CMJ) test in elite youth soccer players.

	Pre-match Mean (SD)	Post-match Mean (SD)	*t* test (*p*)	Change in mean (95% CI)	ES Hedges's *g* (95% CI)		TE × 1.5	Sensitivity to change
Isometric posterior chain test								
Peak Force (N)—Dominant leg at 90°	302.2 (46.9)	272.6 (52.0)	0.002**	−29.6 (−46.2 to −12.9)	−0.58 (−1.44 to 0.29)	M	14.5	Yes
Peak Force (N)—Non-dominant leg at 90°	289.1 (42.6)	259.5 (54.0)	0.005**	−29.6 (−47.9 to −11.2)	−0.59 (−1.45 to 0.28	M	18.4	Yes
Peak Torque (Nm)—Dominant leg at 90°	120.7 (19.9)	109.1 (22.6)	0.003**	−11.6 (−5.0 to −18.2)	−0.53 (−1.39 to −0.34)	M	5.5	Yes
Peak Torque (Nm)—Non-dominant leg at 90°	115.5 (18.8)	103.8 (23.5)	0.005**	−11.7 (−4.3 to −19.1)	−0.53 (−1.39 to −0.33)	M	7.2	Yes
Peak Force (N)—Dominant leg at 30°	–	–	–	–	–	–	–	–
Peak Force (N)—Non-dominant leg at 30°	–	–	–	–	–	–	–	–
Peak Torque (Nm)—Dominant leg at 30°	–	–	–	–	–	–	–	–
Peak Torque (Nm)—Non-dominant leg at 30°	–	–	–	–	–	–	–	–
Countermovement jump test								
Jump Height (cm)	39.5 (4.6)	38.4 (5.0)	0.005**	−1.1 (−1.9 to −0.4)	−0.22 (−1.03 to 0.59)	S	1.4	No
Contraction Time (ms)	692.1 (76.7)	720.9 (89.1)	0.236	28.8 (−21.5 to 79.2)	0.34 (−0.48 to 1.15)	S	46.4	No
RSImodified (m/s)	0.58 (0.11)	0.55 (0.11)	0.013*	−0.03 (−0.06 to 0.01)	−0.26 (−1.08 to 0.55)	S	0.03	Yes
Concentric Duration (ms)	244.2 (31.6)	245.9 (30.6)	0.756	1.7 (−9.8 to 13.1)	0.05 (−0.76 to 0.86)	T	17.8	No
Concentric Peak Force (N)	1,843.2 (185.1)	1,765.7 (181.5)	0.008**	−77.5 (−130.5 to −24.5)	−0.41 (−1.23 to 0.41)	S	86.3	No
Concentric Peak Velocity (m/s)	2.86 (0.15)	2.82 (0.17)	0.051	−0.04 (−0.07 to 0.00)	−0.24 (−1.05 to 0.57)	S	0.06	No
Concentric Mean Power (W)	2,239.3 (315.4)	2,141.2 (290.1)	0.000***	−98.1 (−142.2 to −54.0)	−0.31 (−1.13 to 0.50)	S	87.9	Yes
Peak Power (W)	3,981.9 (523.7)	3,943.9 (483)	0.282	−38.0 (−111.6 to 35.6)	−0.07 (−0.88 to 0.74)	T	141.0	No
Eccentric Duration (ms)	447.8 (56)	475.1 (66.2)	0.173	27.2 (−13.7 to 68.2)	0.43 (−0.39 to 1.25)	S	29.0	No
Eccentric Braking Phase Duration (s)	0.27 (0.05)	0.29 (0.05)	0.212	0.02 (−0.01 to 0.05)	0.35 (−0.47 to 1.16)	S	0.03	No
Eccentric Deceleration Phase Duration (s)	0.15 (0.03)	0.17 (0.03)	0.089	0.02 (−0.00 to 0.04)	0.65 (−0.19 to 1.48)	M	0.02	No
Eccentric Mean Braking Force (N)	888.6 (77.5)	850.3 (75.1)	0.025*	−38.3 (71.0 to −5.6)	−0.49 (−1.31 to 0.34)	S	32.7	Yes
Eccentric Mean Deceleration Force (N)	1,344.7 (151.1)	1,229.9 (133.2)	0.002**	−114.8 (−178.7 to −50.9)	−0.78 (−1.62 to 0.06)	M	74.0	Yes
Eccentric Peak Force (N)	1,769.8 (213.1)	1,621.2 (194.7)	0.000***	−148.6 (−205.4 to −91.8)	−0.71 (−1.54 to 0.13)	M	97.2	Yes
Force at Zero Velocity (N)	1,761 (215.2)	1,613.6 (194.4)	0.000***	−147.4 (−204.9 to −89.8)	−0.70 (−1.53 to 0.14)	M	99.4	Yes
Eccentric Mean Power (W)	487.5 (76.7)	442.1 (65.9)	0.005**	−45.5 (−74.0 to −16.9)	−0.61 (−1.44 to 0.22)	M	34.3	Yes

CI, confidence intervals; TE, typical error of measurement; ES, effect size; T, trivial; S, small; M, moderate, RSI, reactive strength index.

Sensitivity to change: absolute change at post-match >TE x 1.5 (Hopkins, 2000).

* *p* ≤ 0.05.

** *p* ≤ 0.01.

*** *p* ≤ 0.001.

## 4. Discussion

The purpose of this study was to quantify the reliability and examine the sensitivity to change of a range of physical performance measures in a group of elite youth soccer players. The main findings from this study were: (1) IPC and CMJ neuromuscular measures demonstrated a high level of absolute reliability (CVs from 1.5 to 8.8%) and *moderate* to *excellent* level of relative reliability (ICCs from 0.70 to 0.98); (2) IPC peak force and torque, CMJ RSImod, CMJ eccentric forces (EccMBF, EccMDecF, EccPF, F@0 V) and CMJ mean power measures showed significant reductions of *small* to *moderate* effect and acceptable sensitivity by displaying post-match changes greater than their typical variation (Figures 2, 3); and (3) CMJ concentric phase measures and jump height showed *trivial* to *small* alterations by displaying post-match changes similar to their typical variation in elite youth soccer players (Figures 2, 3). These findings suggest that IPC peak force and torque, CMJ RSImod, CMJ eccentric phase forces and CMJ mean power may be considered more suitable measures for monitoring players’ post-match neuromuscular performance in elite youth soccer players.

### 4.1. Reliability of physical performance measures

IPC lower-limb muscle peak force and torque were demonstrated to be reliable for both dominant and non-dominant leg at 90° and 30° knee flexion. There is no direct comparative reliability data reporting peak torque recorded during the IPC test in youth soccer players. However, our findings are consistent with previous literature on professional soccer players which have reported better repeatability at 90° knee flexion compared to 30° knee flexion position (CV: 4.3% and 6.3%, respectively) (34). When comparing our reliability data with that of peak force from previous studies (34, 35), it appears that peak torque has greater absolute and relative reliability (CV <5%; ICC >0.90) compared to peak force. One important consideration in reporting peak torque rather than peak force data is that it accounts for individual differences in limb length (42). Practitioners may therefore consider the IPC as a reliable neuromuscular performance test for monitoring post-match response in youth soccer players.

Most of the CMJ measures from the eccentric and concentric phases demonstrated a high level of absolute and relative reliability in our sample. Previous literature in youth soccer quantified the reliability of jump height only (19, 20). Our results showed greater absolute (CV: 2.6%) and relative (ICC: 0.93) reliability for jump height. These differences may be a result of the different technologies used between studies (i.e., force platform vs. contact mat vs. optical system). Additionally, we quantified the reliability of a wide range of eccentric and concentric phase measures which may provide deeper insights related to both jump outcome and movement strategy (28). Our CVs are lower than in previous literature that used a similar methodology (28, 29), which may be due to the differences in testing protocols, environment and population. However, CVs (1.7%–11.0%) and ICCs (0.70–0.99) were similar to that of a recent study conducted with professional rugby union players which quantified the reliability of 86 CMJ measures (16). Interestingly, CMJ concentric phase and jump outcome measures (e.g., jump height and peak power) displayed better reliability statistics when compared with time-based CMJ measures, suggesting greater variability of jump strategy measures. Despite this trend, several jump strategy measures (e.g., EccDur, RSImod, F@0 V) demonstrated an acceptable level of reliability and therefore may be valuable for monitoring post-match physical performance. In this light, this study uniquely provides reliability reference data of a comprehensive range of CMJ measures within an elite youth soccer academy environment.

### 4.2. Sensitivity of physical performance measures

Determining the inclusion or exclusion of neuromuscular performance measures based on reliability alone may lead to erroneous conclusions. A novel aspect of the present study was to establish the level of reliability in conjunction with the sensitivity to change for effectively monitoring post-match neuromuscular performance in youth soccer players. The significant reductions in IPC measures at 90° dominant and non-dominant leg (−10% at post-match) suggest that soccer match play results in acute alterations of hamstring neuromuscular function in youth soccer players, in agreement with previous literature (36). However, the magnitude of post-match decline recorded in our research is smaller compared to previous studies (−15% at post-match) that assess hamstring neuromuscular function following a 90 minute competitive match (34, 43). Despite the difference in the magnitude of change, IPC peak force and torque were demonstrated to be sensitive to change by detecting post-match changes greater than the typical variation for both dominant and non-dominant legs. These moderate reductions in force and torque (−10% at post-match) likely reflect the high eccentric involvement of the hamstring muscles during repeated sprinting, which could alter muscle function and lead to exercise-induced muscle damage in the days following the competition (44). Considering that neuromuscular fatigue is one potential hamstring strain injury risk factor, our findings support the use of IPC test to indirectly monitor post-match neuromuscular function in youth soccer population.

A key finding of the present study was that CMJ eccentric phase measures had greater post-match changes compared with CMJ jump height and concentric phase measures. In line with previous studies on elite youth soccer players (6, 45), jump height showed a significant reduction but demonstrated limited sensitivity by displaying post-match changes similar to the typical variation. It has been reported that jump height may not be sensitive to training and match loads in elite soccer players (26, 46). Therefore, CMJ time-based measures may hold superior sensitivity to change following official matches and fatiguing intermittent protocol (22, 28). Our analysis of CMJ measures revealed that RSImod, eccentric forces (EccMBF, EccMDecF, EccPF, F@0 V) and mean power (ConcMP, EccMP) demonstrated an acceptable level of sensitivity displaying changes greater than the typical variation (i.e., 1.5 times the TE). Gathercole and colleagues demonstrated a similar level of sensitivity to alternative CMJ measures in a group of collegiate team sports athletes showing acute changes greater than their typical variation and confidence intervals (28). The observed responses suggest that elite youth soccer players may experience acute reductions in neuromuscular function which have been attributed to a reduced central drive and metabolic disturbances that impair excitation-contraction coupling and reduce stretch-reflex sensitivity, muscle stiffness, and force production (47). The greater magnitude of change of CMJ eccentric forces may be associated with the frequent intense acceleration and deceleration actions performed during the match (48, 49). Overall, the acute changes in time-based measures may reflect the altered stretch-shortening cycle (SSC) function and movement strategy employed over the downward phase of the CMJ (47). The current findings demonstrate CMJ eccentric force measures may reveal alterations in jump strategy which underline the presence of altered SSC and neuromuscular fatigue following match-play. As time, force and velocity used to perform soccer-specific actions are considered fundamental for success in elite soccer, these alterations in CMJ eccentric force measures may have relevant implications in monitoring players' physical performance.

This study provides practitioners with a range of physical performance measures suitable for indirectly monitoring players' post-match neuromuscular status in elite youth soccer players. It is important to highlight the main limitations inherent to this work. Despite the ecological validity of the study, sensitivity to change was evaluated using a single match of a shorter duration compared to the official match (i.e., 80 vs. 90 min), with match demands inherently variable between players. Thus, match demands might have influenced the magnitude of alterations post-match. Another limitation of our study is the lack of investigation into the association between changes in physical performance and match demands. Examining this association may provide valuable insights for predicting post-match acute and residual fatigue (49). Additionally, it is important to emphasize that our study was conducted with players from one club only, limiting the generalizability of our results to other youth soccer academy settings. Future research should investigate the time course of recovery of these neuromuscular measures in the days following the match and examine the effects of different recovery interventions on these measures in the subsequent days following match play (i.e., match-day + 1 and match-day + 2) as little research exists in elite youth soccer (50).

## 5. Practical applications

The present study demonstrated that IPC and CMJ tests are reliable and sensitive tools for monitoring post-match changes in physical performance of elite youth soccer players. Our findings indicated that IPC peak force and torque, CMJ RSImod, CMJ eccentric measures (EccMBF, EccMDecF, EccPF, F@0 V) and CMJ mean power may be more appropriate to detect physical performance changes following match-play. Conversely, typically reported CMJ measures such as jump height and peak power showed limited sensitivity in detecting post-match changes. Performance practitioners are therefore encouraged to include such measures for monitoring neuromuscular performance following the match. This might allow coaches and scientists to optimize the scheduling of training across the microcycle, facilitating recovery whilst also optimizing the physical conditioning of players.

## Data Availability

The raw data supporting the conclusions of this article will be made available by the authors, without undue reservation.
