# Supplementary material for: Reliability and sensitivity to change of post-match physical performance measures in elite youth soccer players
Source: Front Sports Act Living. 2023 Jul 13;5:1173621. doi: 10.3389/fspor.2023.1173621 (PMC10374287; doi:10.3389/fspor.2023.1173621)
Supplement: Supplementary file 1 [file Table1.docx]

Supplementary Material

The reliability and sensitivity of post-match neuromuscular measures in elite youth soccer players

Alberto Franceschi, Mark A. Robinson, Daniel Owens, Thomas Brownlee*, Duccio Ferrari Bravo, Kevin Enright

*** Correspondence:** Thomas Brownlee: t.brownlee@bham.ac.uk

# Supplementary Table

Supplementary table. Description of the neuromuscular measures analysed from the countermovement jump (CMJ).

|  | | | |
| --- | --- | --- | --- |
| **CMJ neuromuscular measure** | **Unit of measure** | **Abbreviation** | **Description** |
| Jump Height | cm | JH | Maximal jump height computed using flight time equation. |
| Contraction Time | ms | CT | Duration from jump initiation to take-off. |
| RSImodified | m/s | RSImod | Jump height (calculated from flight time) divided by contraction time. |
| Concentric Duration | ms | ConcDur | Duration of the concentric phase (time from zero velocity to take-off). |
| Concentric Peak Force | N | ConcPF | Greatest force achieved during the concentric phase. |
| Concentric Peak Velocity | m/s | ConcPV | Greatest velocity achieved during the concentric phase. |
| Concentric Mean Power | W | ConcMP | Mean power during the concentric phase. |
| Peak Power | W | PP | Maximum power achieved during the concentric phase. |
| Eccentric Duration | ms | EccDur | Duration of the eccentric phase. |
| Eccentric Braking Phase Duration | s | EccBrakPhDur | Period from minimum force to the start of the concentric phase. |
| Eccentric Deceleration Phase Duration | s | EccDecPhDur | Time period from maximum negative velocity to zero velocity at the end of the eccentric phase. |
| Eccentric Mean Braking Force | N | EccMBrakF | Mean force during the eccentric breaking phase from minimum force to zero velocity at the end of the eccentric phase. |
| Eccentric Mean Deceleration Force | N | EccMDecF | Mean force during the eccentric breaking phase from minimum force to zero velocity. |
| Eccentric Peak Force | N | EccPF | Greatest force achieved during the eccentric phase. |
| Force at Zero Velocity | N | [F@0V](mailto:F@0V) | Force exerted at concentric onset. |
| Eccentric Mean Power | W | EccMP | Mean power during the eccentric phase from start of movement to zero velocity. |

RSI: reactive strength index.
